# Constitutional copy number amplifications: rare or under-evaluated? Revisiting a 25-year-old cold case

**DOI:** 10.1038/s41431-025-01883-0

**Published:** 2025-06-04

**Authors:** Eliana Salvo, Romano Tenconi, Roberto Giorda, Sara Bertuzzo, Luca Cesana, Roberta Murru, Sabrina Giglio, Mana M. Mehrjouy, Niels Tommerup, Orsetta Zuffardi, Maria Clara Bonaglia

**Affiliations:** 1 Cytogenetics Laboratory, Scientific Institute, IRCCS Eugenio Medea, Bosisio Parini, Lecco, Italy; 2 Clinical Genetics Unit, Department of Women and Children’s Health, University of Padova, Padova, Italy; 3 Medical Genetics, R. Binaghi Hospital, Cagliari, Italy; 4 Medical Genetics Unit, Department of Medical Sciences and Public Health, University of Cagliari, Cagliari, Italy; 5 Department of Cellular and Molecular Medicine (ICMM), University of Copenhagen, Copenhagen, Denmark; 6 Department of Molecular Medicine, University of Pavia, Pavia, Italy

**Keywords:** Genetics, Cytogenetics

## Abstract

We reanalyzed through a cytogenomics approach a case published 20 years ago, describing a girl with developmental delay and epilepsy. Karyotype and FISH analysis showed a de novo 2.3 Mb terminal inverted-duplication at 8q24.3. The interpretation was inconsistent with the absence of a more distal deletion as expected for distal inverted duplications, and it was inconceivable to highlight rearrangements smaller than 5–10 Mb at that time. Chromosomal microarray (CMA), optical genome mapping (OGM), and short-read whole genome sequencing (srWGS) identified a complex configuration at 8q24.3, which resembles events like chromoanasynthesis or DUP-TRP/INV-DUP (duplication-triplication/inverted-duplication), both characterized by clustered duplications and triplications, some of which are inverted. In the EBV-line genes located in the amplified regions were overexpressed. Despite a more precise definition of the imbalance, we were unable to provide a clear-cut explanation for the proband’s clinical features.

## Introduction

We re-examined the genome of a previously described girl affected by developmental delay and epilepsy [1]. At the age of 6, her karyotype and FISH revealed a de novo inverted duplication (INV-DUP) at distal 8q of 2.3 Mb. Questions remained about how karyotype analysis identified such a small rearrangement and why the distal inv-dup was not associated with deleting a more terminal region, as described in INV-DUP DEL rearrangements [2]. To address the problem, we re-analyzed the genome of the proband on a new blood sample by CMA, srWGS, and OGM. Overall, we detected a complex configuration at 8q24.3, which resembles but does not exactly overlap events like chromoanasynthesis or DUP-TRP/INV-DUP (duplication-triplication/inverted-duplication), both characterized by clustered duplications and triplications, some of which inverted [3, 4]. Genes within amplified regions were overexpressed despite a more precise genomic definition of the imbalance, the etiology of the proband’s clinical features remains unclear, similar to many amplifications reported in sporadic rearrangements.

## Patient and methods

We report the nineteen-year clinical follow-up of a patient’s case published in 2005 [1]. The proposita, currently 25 years old, has been characterized by severe neurodevelopmental delay and unexplained epilepsy since her assessment at age 6. At 9^8/12^ years old, her weight and height were in the 10th and 3rd percentiles, respectively, and her cranial circumference (CC) was -2SD. She showed severe ID, absence of language, and stereotyped movements, along with lower limb hypertonia, equinovarus deformity, skin dryness, and flaking. Tonic-clonic convulsive seizures became more frequent at 12 years of age when menarche appeared. At the age of 25 years, her weight and height were in 10–25th percentile, and CC was 1.03 DS. She exhibited short philtrum and malar hypoplasia, bilateral clinodactyly, right camptodactyly of the fifth finger, and club foot with syndactyly of the second and third right fingers. Stereotyped movements, ataxic gait, and hypertonia were evident. Molecular investigations (CMA, RT-PCR, quantitative expression PCR, srWGS, OGM, Sanger sequencing validation, and parental origin) are described in the supplemental material.

## Results

**Fig. 1:**
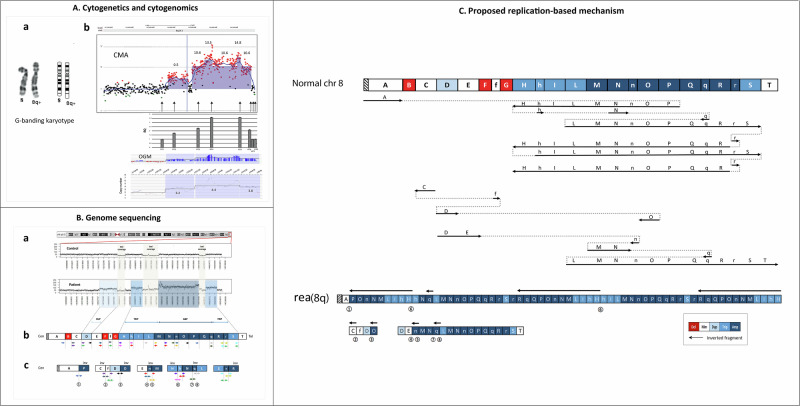
Characterisation and interpretation of the 8q24.3 rearrangement. **A** Cytogenetics and cytogenomics data: **a** Cut-out of chromosome 8 from a 400-banding karyotype; **b** CMA, RT-PCR and OGM of the 8q24.3 region showing nearly identical profiles. Numbers represent the log2 ratio (ADM2) of CMA and fractional copy numbers of OGM. **B** Genome sequencing details (**a**) NGS coverage plot (50x) of the 8q24.3 region (chr8:142,664,805-145,138,635, hg38) in control and the proband showing the duplicated (lighter blue box), triplicated (blue box), and amplified regions (darker blue boc) present in the patient only; quantification of copy number gains was based on the increased coverage in the plot (see Table S1). The three light gray boxes, present both in the control and the proband, indicate portions with poor coverage. **b** Schematic drawing of the order of the shattered fragments, according to hg38. In white, neutral copy number fragments (A, C, E, f, T); in red, deleted fragments (B, F, G); in light blue, the duplicated fragment D; in blue, the triplicated fragments H, h, I, L, S, and in dark blue, the amplified ones M, N, n, O, P, q, R, s. Left or right-oriented arrows represent discordant reads as they appear when aligned back to the reference genome (hg38). Note that hN junction one breakpoint lies within a clusted of segmental duplication (see Table S3) **c** Partial reconstruction of rearranged blocks; numbers within circles correspond to the breakpoint junctions verified by Sanger sequencing analysis (see Fig. S4). **C** The plausible replication-based mechanism of the 8q24.4 rearrangement. The schematic drawing indicates the 8q normal chromosome segmented according to the breakpoints (see panels **B** and Table S1). In the proposed replication pathway, arrows in the normal orientation (from left to right) or inverted (from right to left) denote the 8q24.3 regions shuffled on the patient’s chromosome 8q. The dashed lines indicate the movement from one replication fork to another, either in the normal or reverse orientation. At the bottom is a diagram of the final new rearranged chromosome 8 [rea(8q)], where the distal portion 8q24.3 presents inversions, deletions, duplication, triplication, and amplification. It is not certain where the block of fragments CfDO and DEnMNqLMNnOPQqRrST are inserted.

The 400 G-banding karyotype showed a distal 8q rearrangement. Dual-color FISH highlighted a distal INV-DUP at 8q23, sized 2.3 Mb [1]. Contrary to our expectations for this type of structural variation, no associated terminal deletion was detected. CMA confirmed the rearrangement’s size and identified two clustered copy number gains, one duplicated and the other at least triplicated, separated by a copy-neutral fragment. No additional relevant copy number variants were detected elsewhere. OGM data showed a CNV profile nearly identical to the CMA (Fig. 1a). RT-PCR confirmed copy number amplification (4 to 6) of selected distal regions (RT2-RT6) and the absence of the terminal 8q deletion using probes RT7-RT8 (Fig. 1a, Table S1). Furthermore, quantitative expression analysis of *GRINA* and *PLEC* in amplified regions showed about fourfold and 25-fold overexpression compared to controls, respectively (Fig. S1). srWGS identified 23 fragments from 43 bps to 701 kbs, five indicated by lowercase letters below 626 bps. All fragments were confined to the 8q24.3 region (chr8:143286493-144976557, hg38) (Table S2). Three were deleted (B, F, G, Figs. S2, 3), one duplicated (D), while others were triplicated (H-L and S) or amplified (M-r). Three normal copy fragments (C, E, f) were located upstream of the genomic TRIP-AMP portion, between deleted and duplicated fragments (Fig. 1b). The distal 162 Kbs of 8q portion (fragment T) were in the same orientation as the reference genome. Overall, the duplicated, triplicated, and amplified fragments were intermixed, with some of the triplications and amplifications that were inverted (Fig. 1c, Table S2).

We were able to verify eight of the breakpoints’ junctions, of which two (junctions 4 and 8) involved microhomology of 1–3 bp and six (junctions 1–3 and 5–7) non-templated insertions of 2–19 bps. Also, at breakpoints 4–5 and 7–8, we identified inserts of 106 (fragment n) and 626 bp (fragment q), respectively (Fig. S4, Table S4). We examined the overall distribution of the repeats elements (UCSC RepeatMasker) near the rearrangement’s breakpoints of which 7/17 contained LTR, Alu, DNA repetitions, LINE/AluSx and one segmental duplication. Five protein-coding RefSeq genes (*JRK, TOP1MT, ZC3H3, EPPK1, ARHGAP39*) were disrupted, as confirmed by Sanger sequencing in three of them (*ZC3H3, EPPK1, ARHGAP39*). Of the three putative fusion genes (Table S4), one could not be confirmed. STS- and SNP-parent-of-origin showed that the duplicated/triplicated/amplified regions involved only one of the two maternal alleles (Table S5).

## Discussion

Although most constitutional pathogenic structural aberrations appear as two breakpoint rearrangements, deeper DNA investigations can show unexpected complexity with the involvement of more than two chromosomes or more than two in cis breakpoints [5–7]. In this case, we were surprised that we did not find any deletion downstream of the duplication, as expected for the INV-DUP DELs, the rearrangements deriving from the breakage of mirror dicentric chromosomes [2]. Another concern was that the rearrangement was visible on the 400-band karyotype, although it would only be 2.3 Mb in size if it was an INV-DUP-DEL. CMA answered both questions: the 8q24.3 rearrangement was a duplication/amplification having nothing to do with the INV-DUP-DELs [8]. In addition, the amplification showed that the rearrangement size was not less than 5–10 Mb, which is the threshold for detecting imbalances by the G banding [9]. The amplified region was, indeed, significantly much larger, with a region of ~540 kb amplified at least threefold (~1.6 Mb) and a portion of ~1.14 Mb amplified from at least fourfold (~4.6 Mb) to over eightfold (~9 Mb) (Fig. 1a, Table S2).

**Fig. 2:**
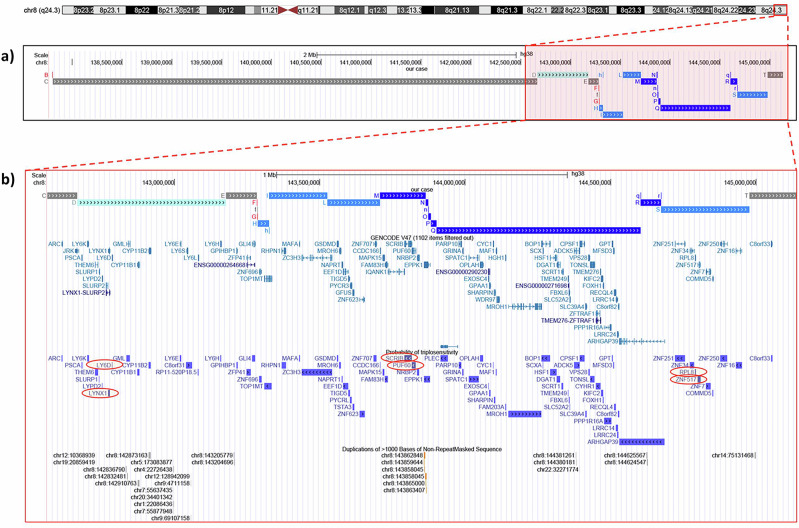
Genome view of the rearranged genomic region 8q24.3 spanning the distal 2.5 Mb of chromosome 8q (GRCh38/hg38). **a** The colored horizontal lines represent the rearranged segments of our patient’s identified by genome sequencing. Each color indicates the copy number status: gray means normal copy numbers; red denotes deletions; light blue indicates duplication; blue represents triplication, and dark blue amplification. **b** Magnified view of the 8q24.3 DUP/TRIP/AMP. From top to bottom: UCSC genes (GRCh38/hg38); triplosensitivity genes (pTriplo) map: in red circles those having a probability of triplosensitivity (pTriplo ≥0.94) [17]; segmental duplications >1000 bps.

WGS partially resolved the variant’s structure, showing a series of contiguous triplicated and amplified fragments, preceded by a duplicated region (D in Fig. 1b, c) of smaller size, which was interspersed between normal copy number segments and copy number losses. The inverted orientation of some of the amplified segments immediately suggested iterative intrachromosomal switches within a clustered chromosomal region. In agreement, genotyping data showed that informative markers (2 / 3) within the amplified regions were from a single maternal chromosome 8. This type of amplification has been reported as a consequence of chromoanasynthesis or DUP-TRP/INV-DUP rearrangements, both in cancers [10, 11] and constitutional diseases [4, 12]. None of these types of rearrangements is recurrent; that is, they do not share the same breakpoints. However, when they insist on the same dosage-sensitive gene, they give rise to specific pathogenic conditions. The *MECP2* locus at Xq28 provides an impressive example: in hemizygous males, a severe developmental disorder (MIM#300260) associates with the triplication of the gene, which in turn is secondary to a DUP-TRP/INV-DUP reorganization. Mapping the breakpoint junctions in several cases [4] has revealed that, despite the overlapping core features associated with *MECP2* amplification [13], the DUP-TRP/INV-DUP structure was diverse in the different cases. In contrast, we could not find cases overlapping the clinical and molecular features of the proband described here. A few cases with 8q24.3 inverted duplications without any associated triplication are reported [14]. Indeed, in one case describing an inv-dup del(8q), the region contiguous to the deletion was partly more than triplicated for about 90 kb [15, 16], suggesting that further sequencing investigations might reveal more complexity. According to Collins et al. 2022 [17], the 8q24.3 region includes six triplosensitivity genes: *LYNX1*, *LY6D*, *SCRIB*, *PUF60*, *RPL8*, and *ZNF517* (Fig. 2). Of these, only *PUF60* is disease-associated (MIM#615583), although evidence of pathogenicity points to haploinsufficiency (https://www.deciphergenomics.org/gene/PUF60/overview/clinical-info). *GPAA1* (MIM#603048), located within the amplified segment Q, is another disease-associated gene (Fig. 2). Biallelic variants of *GPAA1*, primarily missense ones, have been associated with a disorder (MIM#617810) which includes developmental delay and epilepsy as seen in our patient. However, no pathogeneic or likely pathogenic variant in *GPAA1*, which, along with the amplified allele, could be responsible for the proband’s phenotype were detected. Finally, WGS did not find pathogenic SNVs in other disease-associated genes per ACGM guidelines [18]. Potential pathogenic correlations with interrupted or fusion genes (Table S4) could not be excluded.

In summary, the rearrangement’s molecular definition has clarified that it is much more complex than initially highlighted. The presence of several amplified regions, either in direct or reverse orientation, as shown by WGS and OGM (Fig. 1b, c, Fig. S5), immediately pointed to an aberrant DNA replication at the basis of the event. It remains challenging to distinguish whether it is a process of chromoanasynthesis or a DUP-TRP/INV-DUP event, both characterized by clustered duplications and triplications. The presence of short sequence insertions parallels what Zhang and Pellman (2024) [19] reported in a process they termed break-replication/fusion (B-R/F). Accordingly, this B-R/F explains the complexity of the rearrangement patterns, including composite and rapid DNA amplification after chromosome fragmentation.

Interestingly, some breakpoint junctions appear to have formed two times: hH, Sr, Nq, qL. The latter junction, The structure of the hN junction (Table S4) illustrates the role of segmental duplications in forming complex rearrangements [20]. Indeed, one of its breakpoints is located within a cluster of segmental duplications. Unsurprisingly, this region is recognized as a “problematic region” (UCSC hg38). This finding together the multiple amplification, suggests a rolling circle mechanism underlying the formation of this complex genomic rearrangement [21, 22]. In constitutional diseases, complex rearrangements characterized by clustered duplications and amplifications seem to be less common than those mediated by chromothripsis and NAHR. The former, following partial trisomy rescue, can lead to a series of de novo imbalances, including unbalanced translocations, insertions, and supernumerary small marker chromosomes [23, 24]. Conversely, NAHR-mediated rearrangements can result in mirror dicentric chromosomes, which, after breakage, produce INV-DUP DEL rearrangements and simple distal deletions.

One final consideration concerns the difficulty of correlating the genotype to the phenotype in the presence of amplifications. Indeed, most of them are sporadic rearrangements, and their functional effects, especially in embryogenesis, much less in tumors, are not obvious. This condition may hinder the publication of unsolved cases, making such rearrangements appear even rarer.

## Supplementary information

Supplementary files

Supplementary methods

Table S1-S5

## Data Availability

All data generated or analysed during this study are included in this published article [and its supplementary information files].
