# Supplementary material for: Constitutional copy number amplifications: rare or under-evaluated? Revisiting a 25-year-old cold case
Source: Eur J Hum Genet. 2025 Jun 4;33(9):1212–6. doi: 10.1038/s41431-025-01883-0 (PMC12402127; doi:10.1038/s41431-025-01883-0)

**FIGURE S1: Quantitative expression analysis** of the GRINA gene (chr8:143,990,056-143,993,415, hg38) showed approximately four-fold expression in the proband EBV line, compared to four control EBV lines; the PLEC1 gene (chr8:143,915,153-143,973,529, hg38), showed 25-fold overexpression compared to control lines (Figure 2). Both genes are located on one of the more extensively amplified regions of chromosome 8. The HDAC1 gene on chromosome 1 showed less than two-fold overexpression in the proband.

**
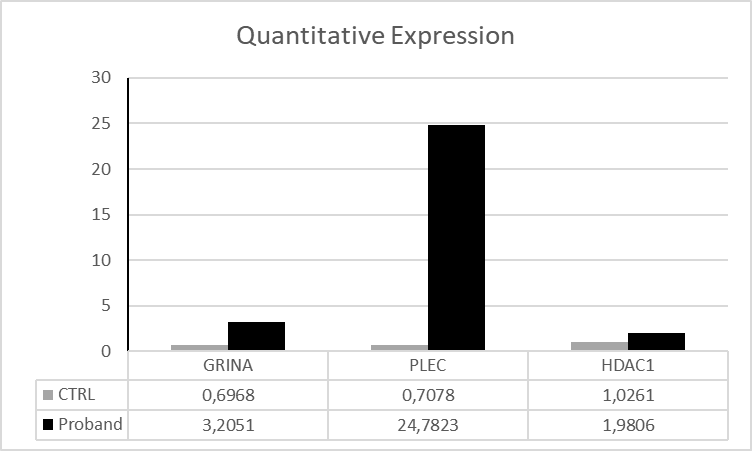
**

**FIGURE S2: deleted fragment B**  IGV illustration indicates the heterozygous 3,2kb deletion at chr8:137,803,073-137,806,291.The deletion was demonstrated by a decreased average coverage of 24.8x in the patient, while the average coverage of whole chromosome 8 was 51.2x. The same region in the control sample was in a balanced state with an average coverage of 52x, while the calculated average coverage of whole chromosome 8 was 53.2x. The discordant reads (green) on both the left and right sides of the deleted portion mapped to chr8:137,803,073 and chr8:137,806,291, respectively, indicating the defection of fragment B.


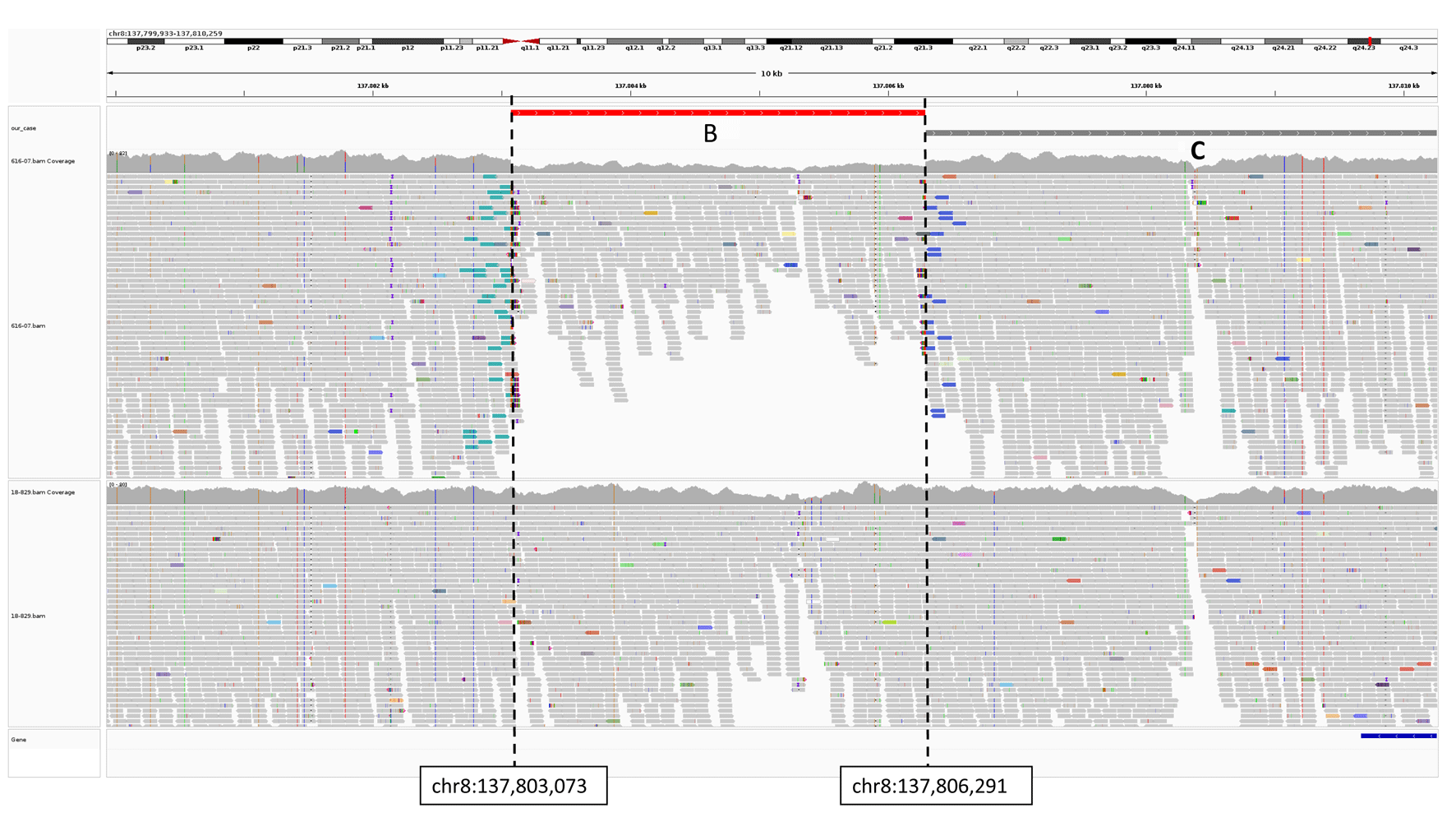


**FIGURE S3: deleted fragments F and G.** IGV illustration indicates the heterozygous deletion of fragment F of 2.5 kb at chr8:143,282,746-143,285,305 and fragment G of 1kb at chr8:143,285,436-143,286,493. The deletion was demonstrated by a decreased average coverage of 24.8x in the patient, while the average coverage of whole chromosome 8 was 51.2x. The same region in the control sample was in a balanced state with an average coverage of 52x, while the calculated average coverage of whole chromosome 8 was 53.2x. The discordant reads (green) on both the left and right sides of the deleted portion mapped to chr8:137,803,073 and chr8:137,806,291, respectively, indicating the defection of fragment B.

**
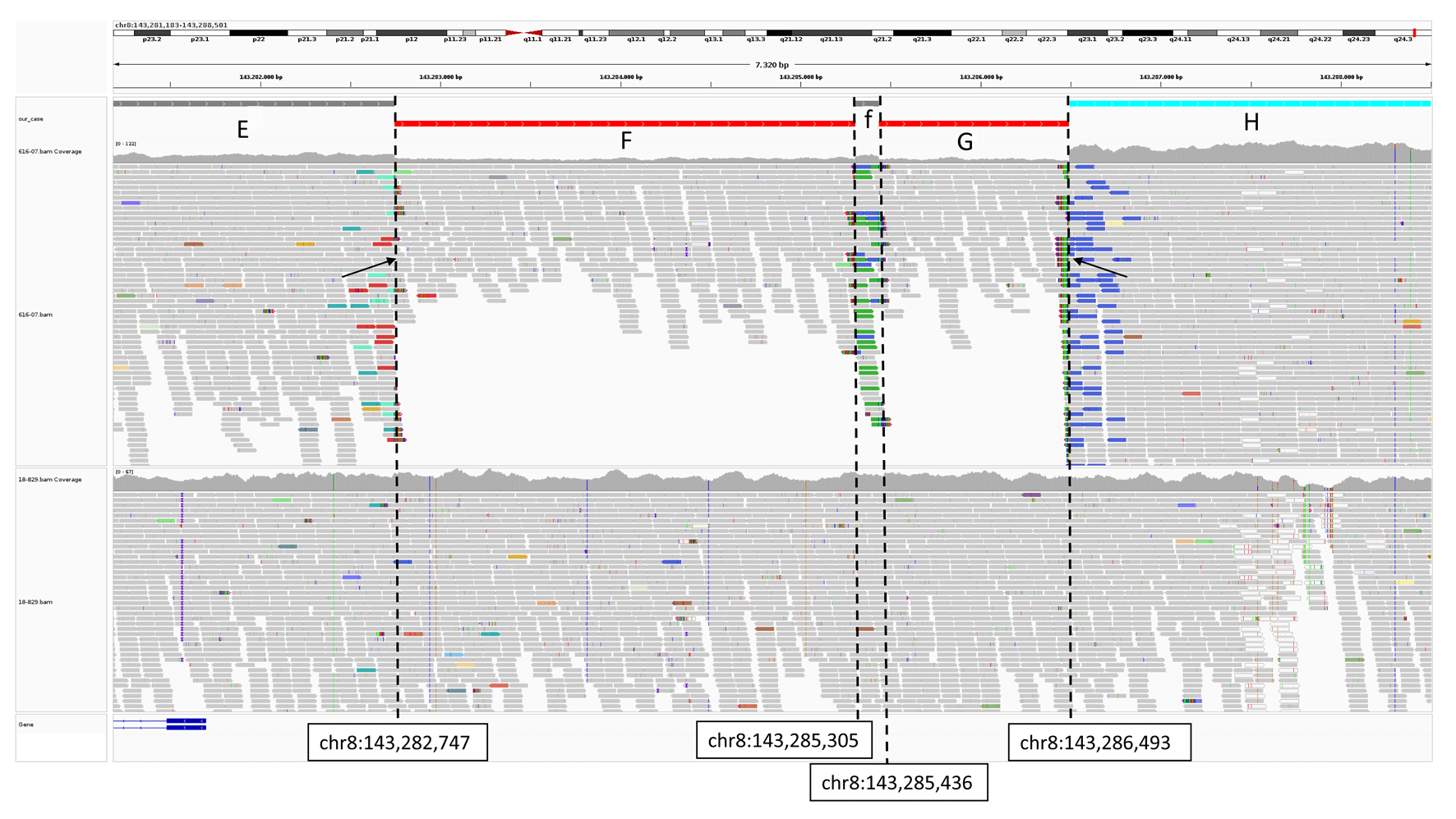
**

**Figure S4**: **Detailed characteristics of all breakpoint junctions confirmed at the nucleotide level by Sanger sequencing (see also Table S3). Top:** : **P:** Proband; **M:** Mather; **F:** Father; **NC:** Normal Control; (-): Negative control). Reference sequences are named (+) plus, (-) minus, to indicate the orientation of the DNA strand. Short microhomologies and insertions found at the breakpoints are boxed in green and red, respectively. In breakpoints 4-5 and 7-8, novel sequence insertions of 106 bp (fragment n) and 626 bp (fragment q), respectively, were identified at the fusion point


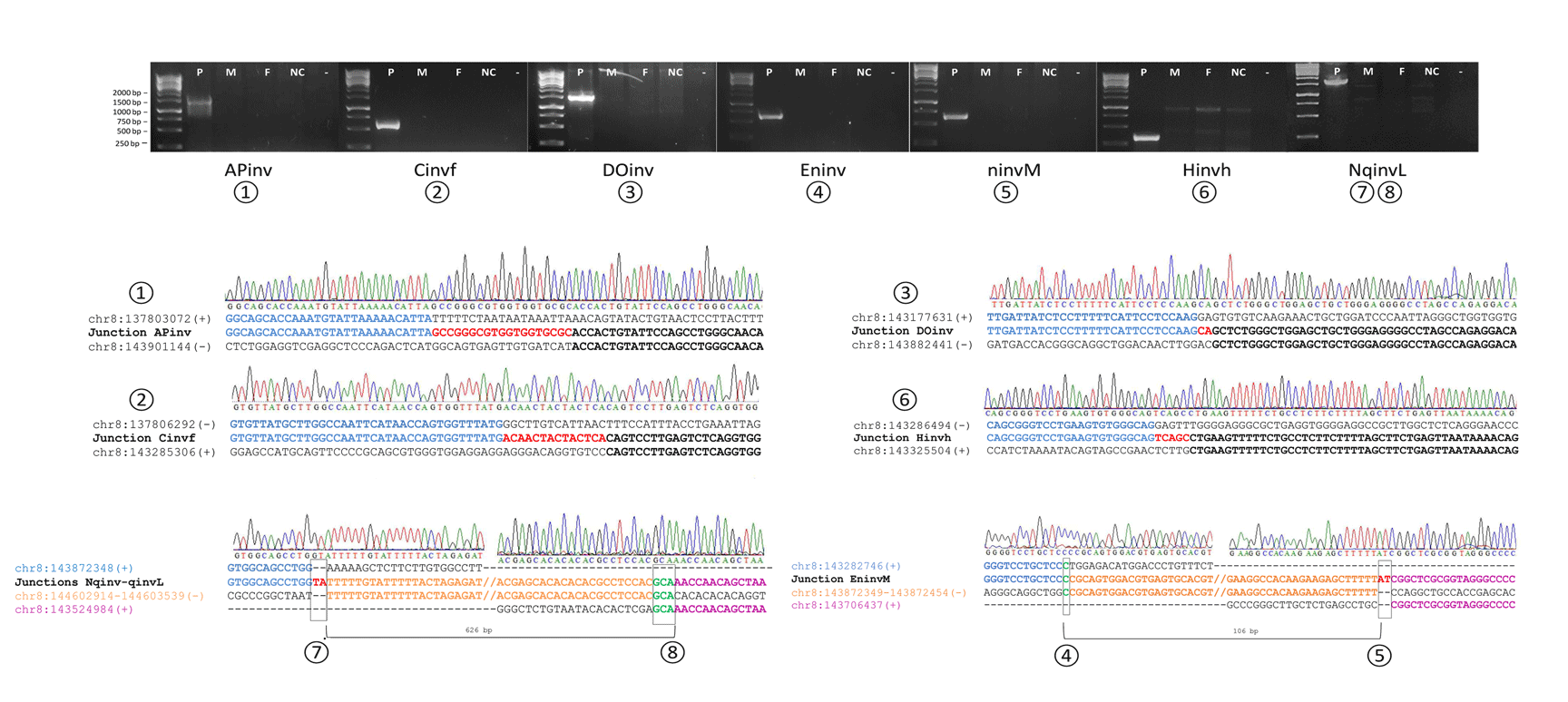


**Figure S5** **OGM: genomic map overview of the 8q24.3 rearranged region**

**Upper** :Copy number (CN) track showing the 8q24.3 DUP-TRIP-AMP (blue square) picked up by the copy number algorithm (See also figure 1A).

**Bottom:** Alignments between reference maps of chromosome 8 (Ref 8) and sample maps (blue bars) are shown as gray strings that indicate the presence of several intrachromosomal fusion and inverted segments.


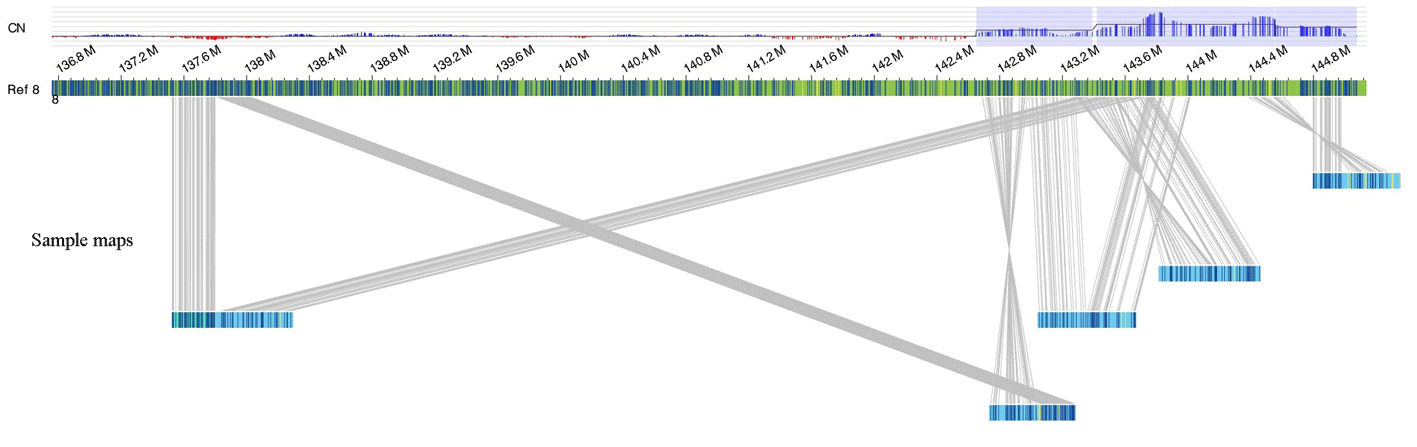

Supplement: Supplementary file 1 — Supplementary files [file 41431_2025_1883_MOESM1_ESM.docx]
