# Supplementary material for: Constitutional copy number amplifications: rare or under-evaluated? Revisiting a 25-year-old cold case
Source: Eur J Hum Genet. 2025 Jun 4;33(9):1212–6. doi: 10.1038/s41431-025-01883-0 (PMC12402127; doi:10.1038/s41431-025-01883-0)
Supplement: Supplementary file 2 — Supplementary methods [file 41431_2025_1883_MOESM2_ESM.docx]

**Supplementary methods**

## **Molecular studies**

***Chromosomal microarray analysis (CMA)***

Genomic DNA was extracted from proband’s and parents’ blood with standard protocols. CMA was performed with Agilent Techonologies array-CGH kit 244A and analyzed via Agilent Cytogenomics V.5.2.0.20. All nucleotide positions refer to the UCSC Genome Browser on Human Genome (GRCh38/hg38)

***Optical genome mapping (OGM)***

The OGM was conducted using ultra-high molecular weight DNA samples (> 150 kb) extracted from the peripheral blood cells of the proband and her parents using an SP Cryopreserved Cell isolation kit (Bionano Genomics, San Diego, California, United States) as previously reported [1].

gDNA was labeled with a direct label (DL) and Stain DNA Labeling Kit using Direct Label Enzyme 1 (DLE-1) and DL-green fluorophores, loaded on a nanochannel chip, and analyzed on a Saphyr instrument (Bionano Genomics). A minimum of 320 Gb of data were acquired. De novo genome map assembly was performed using Bionano Solve software V.3.7. SVs (based on the assembled genome maps) and CNVs (based on molecular coverage) were called against the human reference genome (GRCh38/hg38). Analysis of these data was performed with Bionano Acces V 1.7.0 s and Bionano tools on the Saphyr Compute On Demand server. The following filtering confidence thresholds were applied: insertion/deletion; 0, inversion; 0.7, duplications; −1, intratranslocation; −1 and 0.05, intertranslocation; −1 and 0.05, and CNV; 0.99. A masking filter was applied. For CNV_calls, only segments >500 kb were considered. SVs_calls were filtered using Bionano’s human control sample SV database containing variants collected from > 300 human genomes with no reported disease phenotypes. Only SVs below 1% were taken into consideration.

***RT-PCR***

Eight specific chromosome 8qtel target sequences for Real-Time PCR analysis (RT1 to RT8) were selected within non-repeated portions of the chromosome using Primer Express 3.0 software (Applied Biosystems, Foster City, CA); a control amplicon was selected with the same parameters in the *MAPK1* gene on 22q11. The primers sequence are listed in Table S1. We performed amplification and detection on a ABI PRISM 7900 Sequence Detection System (Applied Biosystems) using SYBR Green PCR Master Mix (Applied Biosystems) as described (Bonaglia et al, 2009).

***Quantitative expression analysis***

Total RNA was extracted from 5x10^6^ EBV-immortalized cells using the Rneasy Mini Kit (Qiagen) according to the manufacturer’s protocol; cDNA was synthesized using the Super Script III First-Strand Synthesis System (Invitrogen). Expression was assessed by Real-Time Quantitative PCR (RT-Q-PCR) on a 7900HT Sequence Detection System (Applied Biosystems) using the following TaqMan Gene Expression Assays (Applied Biosystems) and manufacturer’s protocols: human *GRINA* (Hs00418129_g1), *PLEC1* (Hs00356977_m1), *HDAC1* (Hs00606262_g1), *GAPDH* (Hs99999905_m1).

***Pair-end whole genome-sequencing (PE-WGS)***

Genomic DNA from the proband's and parents' blood was sequenced using the Illumina Hiseq 2000 platform, employing a 30× PCR-free paired-end WGS protocol. Reads from the fastq files were mapped to the human reference genome GRCh38/hg18 using Isaac Genome Alignment Software (V.iSAAC-03.16.06.06)[2]. Coverage graphs were performed plotting the average coverage of 1000 bp windows sliding over the whole chromosome length, in order to identify large deletions and duplications in each chromosome. Structural variants were called using Lumpy (V.0.2.12) and Manta (V.0.29.6)[3-4].

***Mate-pair whole genome sequencing (MP-WGS)***

Mate-pair libraries were constructed using 1 µg of DNA following the instruction for a gel-free preparation of a 2 kb effective insert size library (Mate-Pair Library v2, Illumina) and sequenced on a NextSeq 2000 (Illumina, San Diego, CA, USA). Reads were mapped to the human reference genome GRCh38/hg38 using BWA (Li & Durbin, 2009). The BAM files (containing all the reads, both concordant and discordant) were uploaded into Integrative Genomics Viewer (IGV) (Broad Institute, Cambridge, MA, USA) and the estimated region detected by CMA was used to find the approximate breakpoint regions.

***Data analysis of WGS***

The breakpoints of predicted structural variants were manually checked in Integrative Genomics Viewer (IGV) (Broad Institute, Cambridge, MA, USA) genome browser, and the rearranged chromosome 8 was reconstructed according to the orientations of discordant paired reads detected on each breakpoint.  We could visualize the genomic imbalances related to the DUP/TRIP/AMP using the depth of coverage of the aligned mate-pair and pair-end, together with the cluster of reads that indicated the breakpoint regions. The average read depth was calculated by excluding the read mapping within a region with low-quality coverage (Table S1). Our case was compared with controls to identify potential deletions or duplications.

***Validation of the breakpoint junction identified by WGS***

Segment junctions were confirmed by PCR and Sanger sequencing using primers listed in Supplementary Table S2 primers.

***Parental origin analysis***

We genotyped the family trio either by amplification with primers labeled with fluorescent probes (ABI 5-Fam, Hex, and Tet) followed by analysis on an ABI 310 Genetic Analyzer (Applied Biosystems, Monza, Italy) or SNP array (Human Omni Express Exome ILLUMINA v1.2), as in our previous report [5, 6] (Table S4).

**References**

1. Bonaglia MC, Salvo E, Sironi M, Bertuzzo S, Errichiello E, Mattina T, Zuffardi O. Case Report: Decrypting an interchromosomal insertion associated with Marfan's syndrome: how optical genome mapping emphasizes the morbid burden of copy-neutral variants. Front Genet. 2023 Sep 21;14:1244983. doi: 10.3389/fgene.2023.1244983.
2. Raczy C, Petrovski R, Saunders CT, Chorny I, Kruglyak S, Margulies EH, Chuang HY, Källberg M, Kumar SA, Liao A, Little KM, Strömberg MP, Tanner SW. Isaac: ultra-fast whole-genome secondary analysis on Illumina sequencing platforms. Bioinformatics 2013;29:2041–3. Doi:10.1093/bioinformatics/btt314
3. Layer RM, Chiang C, Quinlan AR, Hall IM. LUMPY: a probabilistic framework for structural variant discovery. Genome Biol 2014;15:R84 R84-2014-15-6-r84 doi: 10.1186/gb-2014-15-6-r84 [DOI]
4. Chen X, Schulz-Trieglaff O, Shaw R, Barnes B, Schlesinger F, Källberg M, Cox AJ, Kruglyak S, Saunders CT. Manta: rapid detection of structural variants and indels for germline and cancer sequencing applications. Bioinformatics 2016;32:1220–2. Doi: 10.1093/bioinformatics/btv710
5. Bonaglia MC, Giorda R, Tenconi R, Pessina M, Pramparo T, Borgatti R, Zuffardi O. A 2.3 Mb duplication of chromosome 8q24.3 associated with severe mental retardation and epilepsy detected by standard karyotype. Eur J Hum Genet. 2005 May;13(5):586-91. doi: 10.1038/sj.ejhg.5201369.
6. Bonaglia MC, Kurtas NE, Errichiello E, Bertuzzo S, Beri S, Mehrjouy MM, Provenzano A, Vergani D, Pecile V, Novara F, Reho P, Di Giacomo MC, Discepoli G, Giorda R, Aldred MA, Santos-Rebouças CB, Goncalves AP, Abuelo DN, Giglio S, Ricca I, Franchi F, Patsalis P, Sismani C, Morí MA, Nevado J, Tommerup N, Zuffardi O. De novo unbalanced translocations have a complex history/aetiology. Hum Genet. 2018 Oct;137(10):817-829. doi: 10.1007/s00439-018-1941-9. Epub 2018 Oct 1. PMID: 30276538.
